# *De Novo* sphingolipid synthesis is essential for *Salmonella*-induced autophagy and human beta-defensin 2 expression in intestinal epithelial cells

**DOI:** 10.1186/s13099-016-0088-2

**Published:** 2016-02-18

**Authors:** Fu-Chen Huang

**Affiliations:** Department of Pediatrics, Kaohsiung Chang Gung Memorial Hospital and Chang Gung University College of Medicine, 123, Ta-pei Road, Niao-sung District, Kaohsiung, Taiwan

**Keywords:** Sphingolipid, *Salmonella*, Autophagy, Human beta-defensin 2, Intestinal epithelia

## Abstract

**Background:**

Sphingolipids are important for innate immune response to eliminate infected pathogens and involved in autophagy. On the other hand, nucleotide-binding oligomerization domain-containing protein 2 (NOD2) served as an intracellular pattern recognition receptor to enhance host defense by inducing autophagy and the production of antimicrobial peptides, such as human beta-defensin-2 (hBD-2). However, the role of sphingolipids in *Salmonella*-induced autophagy and hBD-2 response in intestinal epithelial cells has not been previously elucidated.

**Methods:**

*Salmonella typhimurium* wild-type strain SL1344 was used to infect SW480, an intestinal epithelial cell. hBD-2 and interleukin-8 (IL-8) mRNA expressions were assessed in SW480 cells using RT-PCR, and intracellular signaling pathways and autophagy protein expression were analyzed by Western blot in SW480 cells in the presence or absence of inhibitors or transfected with siRNA.

**Results:**

We demonstrated that inhibition of *de novo* sphingolipid synthesis repressed the membrane recruitment of NOD2 and autophagy-related protein 16-like 1 (Atg16L1), suppressed *Salmonella*-induced autophagic protein LC3-II expression, and reduced NOD2-mediated hBD-2 response in *Salmonella*-infected SW480 cells. Contrasting to the utilization of membrane cholesterol on maintenance of *Salmonella*-containing vacuoles and anti-inflammation by *Salmonella*, sphingolipids act on epithelial defense against the invasive pathogen.

**Conclusions:**

Our results offer mechanistic insights on the role of *de novo* sphingolipid synthesis in the innate immunity of intestinal epithelial cells to *Salmonella* infection. The pharmaceuticals enhancing or diet enriched with sphingolipids may induce the dual anti-bacterial mechanisms. The role of *de novo* sphingolipid synthesis on inflammatory bowel disease is deserved to be further investigated.

**Electronic supplementary material:**

The online version of this article (doi:10.1186/s13099-016-0088-2) contains supplementary material, which is available to authorized users.

## Background

*Salmonella* spp. remain a major public health problem for the whole world. A better understanding of host defense mechanisms of these food-borne pathogens is a prerequisite to design efficient strategies that could reduce the use of antimicrobial agents and drug-resistant *Salmonellosis*.

Recent studies highlight the importance of sphingolipids in regulation of bacterial infections [1, 2]. Sphingolipids are important for innate immune response to eliminate infected pathogens and play a crucial role in infectious diseases [3]. On the other hand, sphingolipids are involved in the regulation of autophagy [4] and might potentially be novel targets for therapeutic intervention in human diseases [5]. For example, sphingolipid synthesis is involved in autophagy in *Saccharomyces cerevisiae* [6]. Nucleotide-binding oligomerization domain-containing protein 2 (NOD2) recruiting autophagy-related protein 16-like 1 (ATG16L1) to the plasma membrane is critical for the autophagic response to invasive bacteria [7, 8]. Additionally, Voss, et al. reported that NOD2 served as an intracellular pattern recognition receptor to enhance host defense by inducing the production of antimicrobial peptides such as human beta-defensin-2 (hBD-2) [9]. Human beta-defensin-2 an antimicrobial peptide induced in various epithelia (e.g. skin, respiratory tract, digestive tract, and genitourinary tract) upon extracellular as well as intracellular bacterial challenge, exhibits a broad spectrum of antimicrobial activity and has been demonstrated to kill bacteria in vivo [10], suggesting that it is an important in host defense against microbes. Sphingolipids and cholesterol act in concert to form raft nanodomains and contribute to Akt/PKB plasma membrane recruitment and activation [11]. Nevertheless, they did not specify the action of sphingolipids and cholesterol. *Salmonella* protects epithelial cells from apoptosis by activation of Akt [12] to form *Salmonella*-containing vacuoles (SCVs), thus escaping from autophagy [13]. Recently, it was observed plasma membrane cholesterol plays a critical role on *Salmonella*-induced autophagy [14]. However, the effects of membrane sphingolipids on *Salmonella*-induced autophagy and hBD-2 in intestinal epithelial cells (IECs) have not been investigated before. It is mandatory to exploit the exact effects of the membrane sphingolipids in IECs infected by *Salmonella*. In the present work, we examine if membrane sphingolipids play a crucial role on the *Salmonella*-induced autophagy and hBD-2 response in IECs via NOD2.

## Results

### Inhibition of de novo sphingolipid synthesis suppresses autophagy proteins expression of Salmonella-infected SW480 cells

**Fig. 1:**
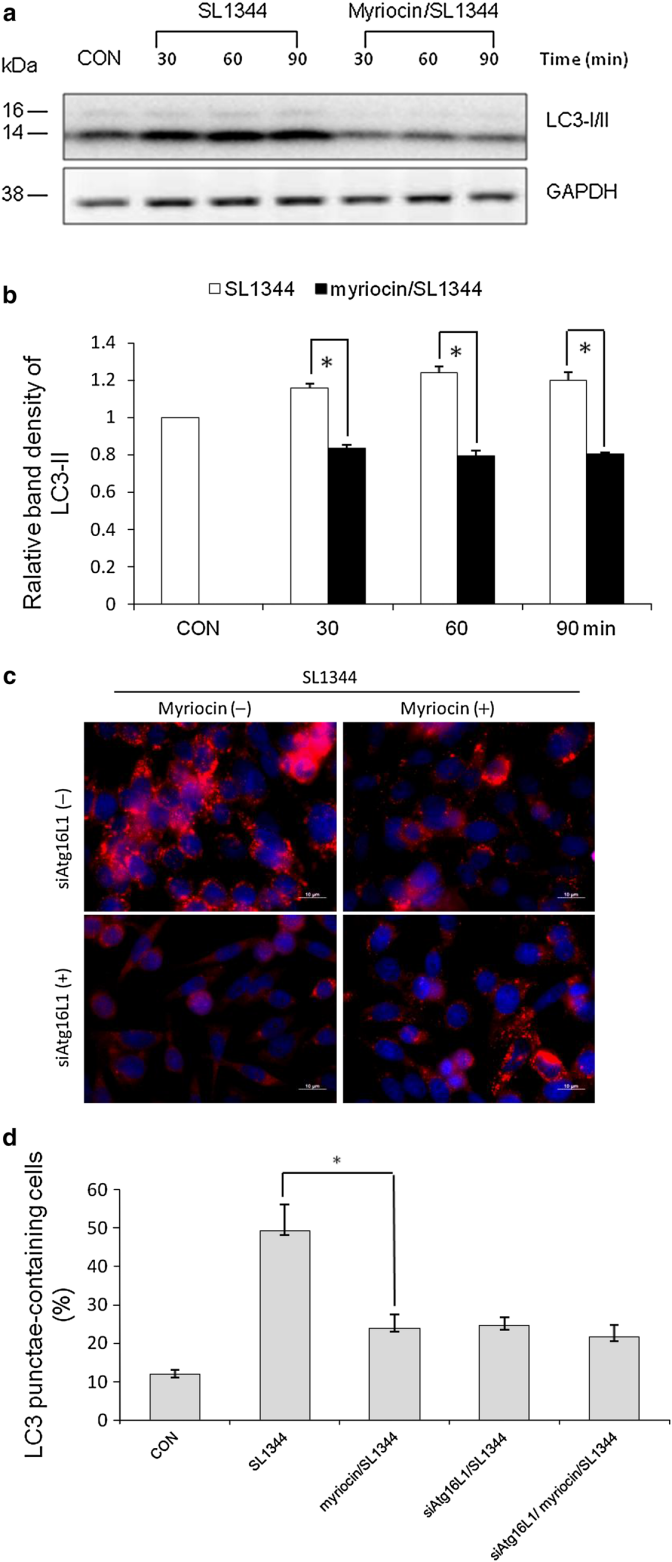
Effect of myriocin on the expression of autophagy in *Salmonella*-infected SW480 cells. SW480 cells were untreated (CON) or treated with myriocin and then infected by *S. typhimurium* wild-type strain SL1344. Immunoblots were performed on whole cell lysates with antibody to detect autophagy LC3-I/II protein expression, or GAPDH for normalization of proteins. Representative immunoblots (**a**) and densitometric quantification of immunoreactive bands are shown. The relative band intensities of LC3-II (**b**) in untreated (*white*) and treated (*black*) SW480 cells are quantified as fold increases compared with the control cells. **c** SW480 cells were transfected with control siRNA or Atg16L1 siRNA (siAtg16L1 (−) = non-target control siRNA; siAtg16L1(+) = siRNA to Atg16L1) for 48 h. The transfected cells were infected for 1 h with *S. typhimurium* wild-type strain SL1344 in the absence or presence of myriocin. The cells were fixed, permeabilized and stained with anti-LC3 (*red*) and LC3 puncta formation was detected by a confocal microscope. Representative images of LC3 punctae are depicted. *Scale bar* = 10 μm. **d** The percentage of cells showing accumulation of LC3 punctae is reported. Each *value* represents the mean ± S.E.M. of 3 independent experiments. An *asterisk* indicates a significant difference (*p* < 0.05)

Sphingolipids are a class of bioactive lipids that mediate many key cellular processes, including apoptosis and autophagy [15]. However, the importance of sphingolipids on *Salmonella*-induced autophagy in intestinal epithelial cells (IECs) has not been reported. To investigate the role of sphingolipids in autophagy proteins expression of IECs infected by *Salmonella*, myriocin which inhibits serine-palmitoyl transferase (the first step in sphingolipid biosynthesis) was used for depletion of sphingolipids. The effectiveness of the inhibitor was confirmed as previously described [16–18]. Myriocin reduced the cellular amounts of glucosylceramide, the major glycosphingolipid in cultured cells, by more than 90 %, as determined by TLC. Overnight starved SW480 cells were untreated or treated by myriocin (10 µM myriocin for 48 h) and then infected by *S. typhimurium* wild-type strain SL1344 for the indicated time. The conversion of LC3-I to LC3-II was detected by Western blot analysis and LC3^+^ autophagosome was analyzed by immunofluorescence. As shown in Fig. 1, *Salmonella*-induced autophagy in SW480 cell was accompanied with an increase in the conversion of LC3-I to LC3-II (Fig. 1a, b) and increased LC3 punctae-containing cells in immunofluorescent analysis (Fig. 1c), while inhibition of sphingolipid synthesis by myriocin suppressed *Salmonella*-induced LC3-II protein and LC3^+^ autophagosome expressions in SW480 cells. It suggests that inhibition of *de novo* sphingolipid synthesis with myriocin suppresses the autophagic process in *Salmonella*-infected SW480 cells. To confirm the suppressive effect of myriocin on autophagy proteins expression, SW480 cells were untreated or treated by myriocin and then infected by *S. typhimurium* wild-type strain SL1344 for the indicated time. The autophagy Beclin-1 and Atg5 proteins expression was analyzed by immunoblot. It was observed that myriocin significantly suppressed *Salmonella*-induced Beclin-1 and Atg5 proteins expression in SW480 cells (Additional file 1: Figure S1).

### Intracellular bacterial count is increased in Salmonella-infected SW480 cells in the presence of myriocin

**Fig. 2:**
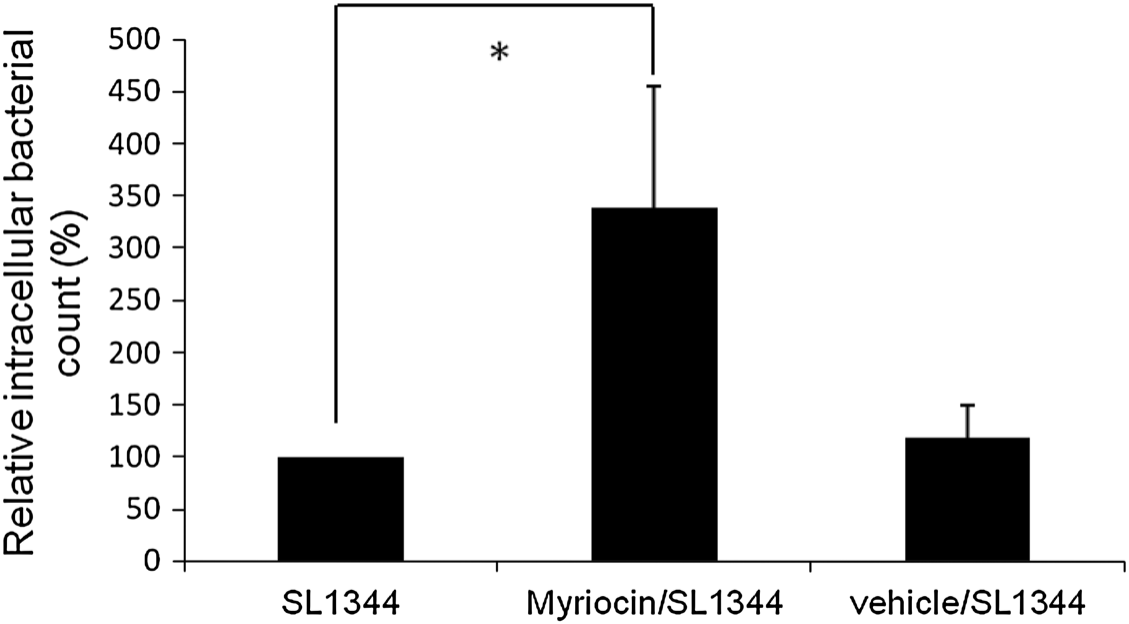
Effect of myriocin on the intracellular proliferation of *S. typhimurium* in cultured IECs. SW480c cells were left untreated, or treated with myriocin or PBS (vehicle), and then infected with wild-type *S. Typhimurium* strain SL1344 and the levels of bacterial proliferation were examined 60 min after infection as indicated in Experimental Section. Lysed cell cultures were plated on LB to count CFUs. *Values* represent the percentage of intracellular bacteria compared with the wild type without any treatment (assigned as 100 %). Each *value* represents the mean ± S.E.M. of 3 independent experiments. An *asterisk* indicates a significant difference (*p* < 0.05)

To investigate if inhibition of *de novo* sphingolipid synthesis suppressed autophagic clearance of the intracellular bacteria in *Salmonella*-infected IECs, SW480 cells were infected by *S. typhimurium* wild-type strain SL1344 in the presence or absence of myriocin. Gentamicin protection assay was performed as in Experimental section. As demonstrated in Fig. 2, myriocin increases the intracellular bacterial count in SW480 cells comparing to the infection only or vehicle-treated cells.

### Inhibition of de novo sphingolipid synthesis suppresses Salmonella-induced membrane recruitment of NOD-2 and Atg16L1 in SW480 cells

**Fig. 3:**
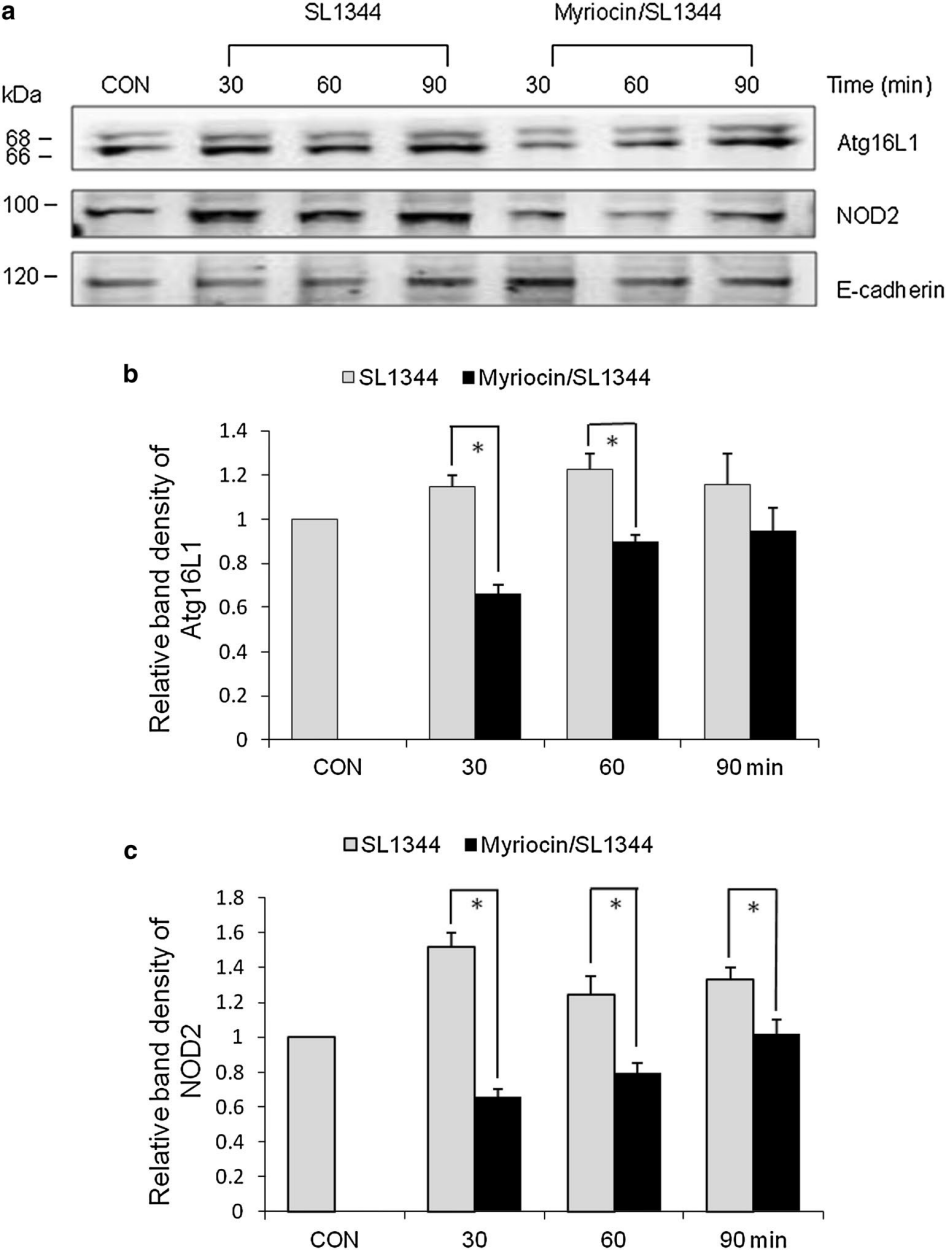
Effect of myriocin on the membrane recruitment of NOD2 and Atg16L1 in *Salmonella*-infected SW480 cells. SW480 cells were untreated (CON) or treated with myriocin and then infected by *S. typhimurium* wild-type strain SL1344. Immunoblots were performed on membrane lysates with antibody to detect Atg16L1 and NOD2expression, and E-cadherin for normalization of membrane protein. Representative immunoblots (**a**) and densitometric quantification of immunoreactive bands are shown. The relative band intensities of Atg16L1 (**b**) and NOD2 (**c**) in untreated (*gray*) and treated (*black*) SW480 cells were quantified as fold increases compared with the control cells. Each *value* represents the mean ± S.E.M. of 3 independent experiments. An *asterisk* indicates a significant difference (*p* < 0.05)

NOD2 is critical for the autophagic response to invasive bacteria because they recruit ATG16L1 to the plasma membrane at the bacterial entry sites [7, 8]. However, the effect of sphingolipid on the membrane recruitment of NOD2 or Atg16L1 is not clear. As demonstrated in Fig. 3, *Salmonella* induced NOD2 and Atg16L1 recruitment into membrane while myriocin suppressed recruitment of Atg16L1 and NOD2 into membrane. Additionally, SW480 cells were observed to have decreased LC3 + autophagosome expression when they were transfected with NOD2 or Atg16L1. It suggests that the decreased recruitment of NOD2 and Atg16L1 to the plasma membrane contributes to the suppressive effect of myriocin on *Salmonella*-induced autophagy in IECs.

### Inhibition of de novo sphingolipid synthesis suppresses Salmonella-induced NOD-2 mediated hBD-2 expression in SW480 cells

**Fig. 4:**
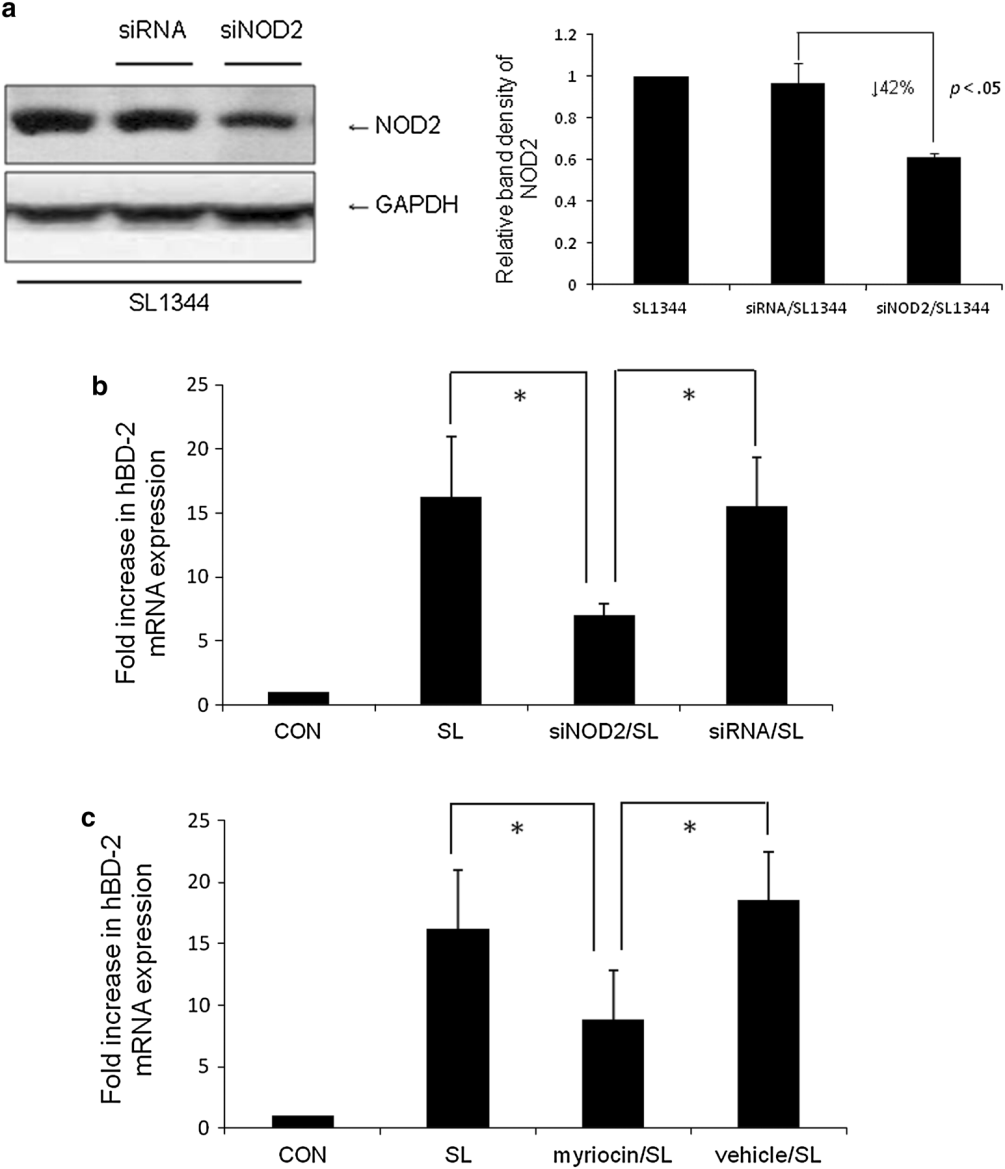
Involvement of NOD2 in the suppressive effect of myriocin on *Salmonella*-induced hBD-2 expression in SW480 cells. **a** Western blots to confirm knockdown of NOD2 in SW480 cells. **b** SW480 cells were transfected with control siRNA or NOD2 siRNA (siRNA = non-target control siRNA; siNOD2 = siRNA to NOD2) for 48 h. The transfected cells were left uninfected (CON) or infected for 1 h with *S. typhimurium* wild-type strain SL1344. **c** SW480 cells were infected by *S. typhimurium* wild-type strain SL1344 in the absence (methanol as vehicle) or presence of myriocin. Total RNA was prepared and analyzed by real-time quantitative PCR to estimate amounts of hBD-2 transcript. The amount of hBD-2 mRNA expression, normalized to the corresponding amount of GAPDH transcript, is shown as the fold increase over uninfected, control cells. *Results* are represented as mean ± S.E.M. for at least three determinations from independent experiments. An *asterisk* indicates a significant difference (*p* < 0.05)

As shown above, inhibition of *de novo* sphingolipid synthesis with myriocin suppressed the membrane recruitment of NOD2. Membrane targeting of NOD2 in intestinal epithelial cells is required for NOD2-dependent NF-κB signaling [19] and subsequent inflammatory responses. NOD2 was reported to induce the production of antimicrobial peptide hBD-2 [9]. It is hypothesized that inhibition of *de novo* sphingolipid synthesis might suppress *Salmonella*-induced NOD2-mediated hBD-2 in IECs. To confirm this hypothesis, two studies were performed. The first, to study NOD2 mediates *Salmonella*-induced hBD-2 response in IECs, SW480 cells were transfected with control nonsilencing siRNA or siRNA targeting NOD2 and infected by *S. typhimurium* wild-type strain SL1344. The uninfected cells worked as control. The knockdown of NOD2 in SW480 cells was demonstrated in Fig. 4a by Western blot (42 %, *p* < 0.05). As illustrated in Fig. 4b, NOD2 siRNA-transfected SW480 cells had significantly less hBD-2 mRNA expression comparing to control siRNA-transfected ones. Secondly, to study if myriocin suppresses *Salmonella*-induced hBD-2 response in IECs, overnight starved SW480 cells were uninfected or infected by *S. typhimurium* wild-type strain SL1344 in the presence or absence of myriocin. As illustrated in Fig. 4c, *Salmonella* SL1344 induces hBD-2 expression in SW480 cells while myriocin suppresses the *Salmonella*-induced hBD-2 mRNA expression. It suggests inhibition of *de novo* sphingolipid synthesis with myriocin blocks *Salmonella*- induced NOD2 recruitment into membrane (Fig. 3) and subsequent suppression of hBD-2 expressions (Fig. 4).

### Inhibition of de novo sphingolipid synthesis results in activation of Akt in Salmonella-infected SW480 cells

**Fig. 5:**
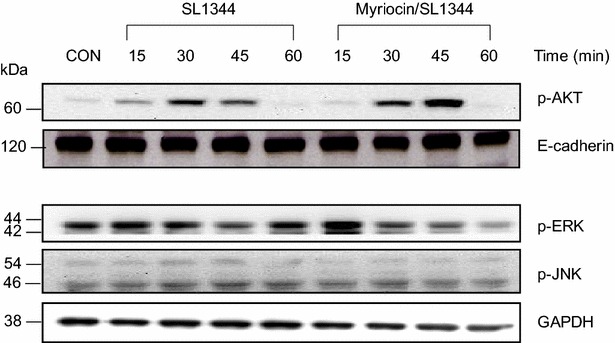
Effect of myriocin on *Salmonella*-activated intracellular signals in SW480 cells. SW480 cells were left untreated (CON), or treated with 10 µM myriocin, and then infected with wild-type *S. Typhimurium* strain SL1344 for the times indicated. Activation of the Akt in cell membrane fraction and ERK and JNK in cytosolic fraction were analyzed by immunoblotting with antibodies to phosphorylated (p) Akt, ERK and JNK. E-cadherin and GAPDH work as normalization of membranous and cytosolic proteins respectively. The *results* shown are representative of 3 separate experiments

Sphingolipids and cholesterol act in concert for the formation of nanodomains [11] that play a crucial role in triggering the phosphatidyl-inositol-3-kinase (PI3 K)/Akt signaling pathway. *Salmonella* protects epithelial cells from apoptosis by activation of Akt [12]. Besides, sphingolipid regulates apoptosis through the activation of extracellular growth factor-regulated kinase (ERK) [20] and JNK [21]. It is reasonable to speculate that membrane sphingolipids may also play a role on *Salmonella*-induced apoptosis in IECs through the modulation of Akt, ERK or JNK signaling pathway. To investigate the role of sphingolipids in activation of Akt, ERK and JNK of IECs infected by *Salmonella*, overnight starved SW480 cells were untreated or treated by myriocin (10 µM myriocin for 48 h) and then infected by wild-type *Salmonella* SL1344 for the time indicated. The activation of the Akt, ERK and JNK was examined by using antibodies specific to the phosphorylated (activated) forms of these proteins. While myriocin had enhanced *Salmonella*-induced phosphorylation of the Akt, it had little or no significant effect on activation of the ERK or JNK (Fig. 5). It suggests inhibition of *de novo* sphingolipid synthesis with myriocin may enhance *Salmonella*-induced anti-apoptosis in IECs via Akt.

### The involvement of PI3 K/Akt in the Salmonella-induced hBD expression after inhibition of de novo sphingolipid synthesis

**Fig. 6:**
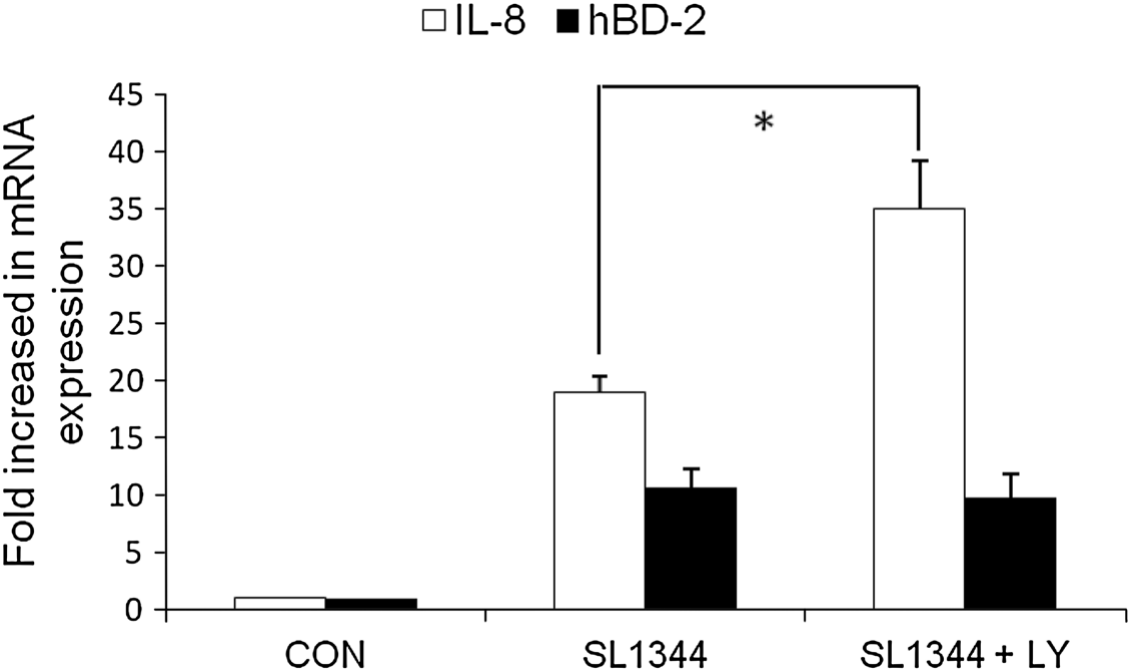
The involvement of PI3 K/Akt on *Salmonella*-induced IL-8 and hBD-2 mRNA expression. SW480 cells were left untreated, or treated with 50 μM LY294002 (LY). They were then infected with the wild-type *S. Typhimurium* strain SL1344 for 1 h. Total RNA was prepared and analyzed by real-time quantitative PCR to estimate amounts of IL-8 and hBD-2 transcript. The amount of IL-8 and hBD-2 mRNA expression, normalized to the corresponding amount of GAPDH transcript, is shown as the fold increase over uninfected, control cells (CON). *Results* are represented as mean ± S.E.M. for at least three determinations from independent experiments. An *asterisk* indicates a significant difference (*p* < 0.05)

It was previously demonstrated that the activation of PI3 K/Akt suppressed *Salmonella*-induced IL-8 response in IECs [22]. To investigate if PI3 K/Akt play the same inhibitory effect on *Salmonella*-induced hBD-2 response, SW480 cells were infected by *S. typhimurium* wild-type strain SL1344 in the presence or absence of LY294002, a PI3 K inhibitor. Total RNA was prepared and analyzed by real-time quantitative PCR to estimate amounts of IL-8 and hBD-2 transcript. The amount of IL-8 and hBD-2 mRNA produced, normalized to the corresponding amount of GAPDH transcript, was shown as the fold increase over uninfected control (CON) cells. As demonstrated in Fig. 6, similar to previous report, LY294002 enhanced *Salmonella*-induced IL-8 mRNA expression while it had no effect on *Salmonella*-induced hBD-2 mRNA expression in SW480 cells. It was further confirmed by the finding that LY294002 did not counteract on the suppressive effect of myriocin on *Salmonella*-induced hBD-2 in SW480 cells (Fig. 6). It suggests that PI3 K/Akt is not involved in the suppressive effect of myriocin on *Salmonella*-induced hBD-2 response in IECs.

To independently corroborate these findings, treatment of Caco-2 cells with fumonisin B1, a fungal mycotoxin that inhibits ceramide-synthetase activity blocking the final step of ceramide synthesis and hence sphingolipid biosynthesis, produced similar effect on the *Salmonella*-induced membrane recruitment of Atg16L1 and NOD2 (Additional file 2: Figure S2) as myriocin without affecting the cell viability. To determine whether the functional consequences of inhibition of membrane sphingolipid was a general phenomenon among different intestinal epithelial cell lines, the effect of FB1 on *Salmonella*-induced hBD-2 expression was examined in Caco-2 cells. Similar results were obtained (Additional file 3: Figure S3).

## Discussion

Recent studies have begun to implicate sphingolipids in regulation of bacterial infections [1, 2]. Increasing evidence indicates the potential of autophagy in controlling infections by directing intracellular or ingested pathogens to lysosomes leading to their destruction [23]. NOD2 and ATG16L1 recruited to the plasma membrane at bacterial entry are critical for the autophagic response to invasive bacteria [7, 8]. The activated autophagy of epithelial cells by NOD2 and Atg16L1 [24] increased killing of *Salmonella* [25]. Inhibition of *de novo* sphingolipid synthesis with myriocin suppressed the membranous recruitment of NOD2 and Atg16L1 (Fig. 3), resulting in reduced autophagic process of *Salmonella*-infected IECs (Fig. 1) and impaired clearance of the intracellular bacteria (Fig. 2). It suggests sphingolipids recruiting NOD2 and Atg16L1 into plasma membrane of IECs infected by *Salmonella* infection contribute to autophagic clearance of invading *Salmonella*. It is compatible with the report [26] that fumonisin B1, a ceramide synthases inhibitor, increases intestinal colonization by pathogenic *Escherichia coli* in pigs. The abnormalities in the handling of intracellular bacteria through autophagy might play a role in Crohn’s disease pathogenesis [24, 27, 28]. The Crohn’s disease associated ATG16L1 coding variant shows impairment of the capture of internalized *Salmonella* within autophagosomes [28]. Studies with experimental animals have shown that feeding sphingolipids inhibits colon carcinogenesis and atherosclerosis [29], suggesting that sphingolipids represent a “functional” constituent of food. The drugs or foods enhancing membrane sphingolipids may induce autophagic clearance of invading pathogens and lower the risk of Crohn’s disease.

NOD2 was reported to serve as an intracellular pattern recognition receptor to enhance host defense by inducing the production of antimicrobial peptide hBD-2 [9]. An increased risk of inflammatory bowel disease (IBD) following enteric infections with *Salmonella* was reported [30]. Dysregulation of NOD2-mediated defense against invasive pathogens may play an important role on the pathogenesis of IBD [31]. Colonic hBD2 was dysregulated at mRNA and protein level in IBD [32]. The antimicrobial dysfunction (e.g. hBD-2) of intestinal epithelial cells to enteric bacteria (e.g. pathogenic adherent-invasive* E. coli* (AIEC)) in patients with NOD2 mutants predispose particularly to ileal involvement in Crohn’s Disease [27]. Children with Crohn’s Disease showed a lower expression of hBD-2 in the inflamed terminal ileum and ascending colon [33]. This study illustrating inhibition of *de novo* sphingolipid synthesis with myriocin suppressed *Salmonella*-induced NOD2-mediated hBD-2 expression (Fig. 4), links the relationship between membrane sphingolipids and inflammatory bowel diseases. In contrast, the synthetic sphingosine analog of myriocin FTY720 leads to a specific down-regulation of proinflammatory signals while simultaneously inducing functional activity of CD4^+^CD25^+^ Treg [34]. FTY720 was suggested to offer a promising new therapeutic strategy for the treatment of IBD. Thus, the role of membrane sphingolipids on inflammatory bowel disease is deserved to be investigated in vivo.

Sphingolipid and cholesterol-based structures called membrane rafts [35], has received much attention in the last few years and are believed to be important structures for the regulation of many biological and pathological processes. These structures attract signaling proteins and allow these proteins to move to new locations for subsequent signaling [36]. It was recently reported [11] that nanodomains play a crucial role in triggering the PI3 K/Akt signaling pathway. However, they did not specify the action of sphingolipids and cholesterol. *Salmonella*-induced cholesterol accumulation in *Salmonella*-containing vacuoles (SCVs) activates PI3 K/Akt signaling [37] and subsequently result in an anti-apoptosis [12] and anti-inflammatory signal [22], both of which may contribute to the invasiveness of *Salmonella* in IECs [37]. As demonstrated in this study that inhibition of *de novo* sphingolipid synthesis with myriocin enhanced the phosphorylation of Akt but suppressed the activation of ERK, membrane sphingolipids in IECs infected by *Salmonella* may play a contrary role on SCV formation to membrane cholesterol [22, 37]. The suppression of phosphorylated Akt may contribute to apoptosis of SCV leading to damaged SCV, which is directed for autophagic clearance. Likewise, although PI3 K/Akt was involved in membrane cholesterol-induced anti-inflammatory response but it is not involved in the effect of depletion of sphingolipids on *Salmonella*-induced hBD-2 expression (Figs. 4c, 6), which is mediated by NOD2 (Fig. 4b). It is very important for the pathogenesis of *Salmonella* infection or innate immunity of the host to defense against the invasive bacteria by regulating the homeostasis between cholesterol and sphingolipids [38]. It is a novel and promising finding that provides therapeutic strategy to enhance sphingolipids beyond cholesterol, in order to enrich innate immunity to *Salmonella* infection.

## Conclusion

Altogether, we demonstrated inhibition of *de novo* sphingolipid synthesis by myriocin, in one way, enhanced the activation of Akt which is important to maintain intact SCV; and in another way, interfered with the recruitment of NOD2 and ATG16L1 into membrane resulting in impairment of autophagy and suppression of hBD-2. It suggests membrane sphingolipids at the entry site of *Salmonella* recruited NOD2 and ATG16L1 to the membrane, inhibit activation of Akt signaling, resulting in the autophagy of the apoptotic SCV and enhanced NOD2-mediated hBD-2 expression. Because manipulating sphingolipids in host cells affects infection and inflammation by the pathogens, pharmacological agents aiming to regulate sphingolipids or diet enriched with sphingolipids [29] could be potentially used in the treatment of infectious or inflammatory bowel diseases in the future.

## Methods

### Reagents

Stock solutions of myriocin and fumonisin B1 (FB1) were prepared as follows: myriocin 1 mg/ml in methanol; FB1, 10 mM in DMSO (all from Sigma, St. Louis, MO).

### Bacterial strain

The wild-type *Salmonella* enterica serovar Typhimurium (*S. Typhimurium*) strain used in this work was SL1344. The preparation of *Salmonella* inoculum has been described previously [14, 39].

### Cell culture and infection

Caco-2, and SW480 cells were purchased from the American Type Culture Collection (Manassas, VA, USA) and were cultured as described previously [14, 37, 39] or as recommended by the manufacturer.

### Inhibition of de novo sphingolipid synthesis

To inhibit sphingolipids synthesis, cells were allowed to attach overnight and were then cultivated in DMEM containing 10 µM myriocin for 48 h as previously described [16]. Cells were stained with Rhodamine 123 (10 μg/ml, 10 min) to confirm that the treatment with inhibitors was not lethal. For lipid analysis, lipids from roughly 2 × 10^6^ cells were extracted and were resolved by two sequential thin layer chromatography (TLC) runs according to Poole et al. [16] with some modification.

### Cell fractionation

Cytosolic and membrane fractions were prepared as previously described [14, 37, 39] with some modification. The protein concentration of extracts was normalized before analysis.

### Western blotting

Equal amounts of protein were analyzed by immunoblot as previously described [14, 37, 39]. The transferred membranes were probed with primary antibodies against phosphorylated (p-)Akt (Cell Signaling, Beverly, MA), (p-)ERK (Santa Cruz Biotechnology, Santa Cruz, CA) or (p-)JNK (New England BioLabs, Beverly, MA), or anti-ATG16L1, LC3B (Cell Signaling, Beverly, MA) or NOD2 (Cayman Chemical, Ann Arbor, MI). After washes, the membranes were incubated with appropriate horseradish peroxidase-associated secondary antibodies before signals were visualized with the enhanced chemiluminescence detection system (Amersham Bioscience).

### hBD-2 assay

After treatment or infection, the culture media were collected and analyzed for hBD-2 by enzyme-linked immunosorbent assay (ELISA) as manufacturer’ s instructions with some modification [40].

### Real-time Reverse Transcription PCR

Total RNA was prepared from control or infected cells. Transcripts were amplified after reverse transcription with random hexamers using the GeneAmp kit (Roche, Nutley, NJ). Real-time reverse transcription-PCR analyses were performed in a fluorescence temperature cycler (LightCycler; Roche Diagnostics) as described previously [14, 37, 39]. For hBD-2, the primers were as follows: forward, 5′-ATCAGCCATGAGGGTCTTGT-3′ and reverse, 5′-GAGACCACAGGTGCCAATTT-3′. For NOD2, the primers were as follows: forward, 5′-AGCCATTGTCAGGAGGCTC-3′ and reverse, 5′-CGTCTCTGCTCCATCATAGG-3′. All quantifications were normalized to the housekeeping gene glyceraldehyde-3-phosphate dehydrogenase. Relative expression is given as a ratio between target gene expression and glyceraldehyde-3-phosphate dehydrogenase expression.

### Immunofluorescence analysis

After infection and treatment, the cultured SW480 cells were fixed, permeabilized and incubated with rabbit anti-LC3B (Cell Signaling Technology, Danvers, MA) (1:250). Secondary antibody was goat anti-rabbit IgG conjugated with Alexa Fluor 594 fluorochrome (Invitrogen Molecular Probes, Eugene, OR, USA). Nuclei were counterstained with fluorescent dye Hoechst (Sigma Aldrich, St. Louis, MO, USA). The percentage of cells with endogenous LC3 punctae was determined by counting the number of positively staining cells from 100 randomly chosen cells from three separate experiments.

### RNA interference (RNAi) in SW480 cells

RNAi experiments in SW480 cells were done as described previously [14, 39], including control nonsilencing small interference RNA (siRNA), NOD2siRNA and different siRNAs targeting Atg16L1 (sequence 1: sense GAGUUGUCUUCAGCCCUGAUGGCAG, antisense CUGCCAUCAGGGCUGAAGACAACUC; sequence 2: sense GGCUCUGCUGAGGGCUCUCUGUAUA, antisense UAUACAGAGAGCCCUCAGCAGAGCC; sequence 3: sense CAAGGGUUCCCUAUCUGGCAGUAAU, antisense AUUACUGCCAGAUAGGGAACCCUUG (sequence were purchased from Invitrogen Corporation, Carlsbad, CA, USA). Briefly, cultured SW480 cells were transfected with chemically synthesized siRNA to silence NOD2 and Atg16L1, respectively. Immunoblotting were performed to examine the efficiency of protein knockdown. For the SW480 cells, 20 nM of each siRNA was transfected 48–96 h before *S. typhimurium* infection.

### Gentamicin protection assay

SW480 cells were pre-treated and infected, then gentamicin protection assay were undertaken as described previously [37].

### Cell viability and morphologic features

Representative cell populations from each condition were examined under light microscopy. No significant change was noted under any condition. Cells were stained with Rhodamine 123 (Santa Cruz Biotechnology, Santa Cruz, CA) to confirm that the treatment with inhibitors was not lethal.

### Statistical analysis

All experiments were carried out at least three times with similar results. Statistical significance was determined using the student’s *t*-test.

### Ethics statement

This was an entirely in vitro study that was approved by the Chang Gung University Biosafety Committee.

## Additional files

10.1186/s13099-016-0088-2 Effect of myriocin on the expression of autophagy in *Salmonella*-infected SW480 cells. SW480 cells were untreated (CON) or treated with myriocin and then infected by *S. typhimurium* wild-type strain SL1344. Immunoblots were performed on whole cell lysates with antibody to detect autophagy Beclin-1 and Atg5 proteins expression, or GAPDH for normalization of proteins. Representative immunoblots (A) and densitometric quantification of immunoreactive bands are shown. The relative band intensities of Beclin-1 (B) and Atg5 (C) in untreated (white) and treated (black) SW480 cells are quantified as fold increases compared with the control cells. Each *value* represents the mean ± S.E.M. of 3 independent experiments. An *asterisk* indicates a significant difference (p < 0.05).
10.1186/s13099-016-0088-2 Effect of fumonisin B1 on the membrane recruitment of NOD2 and Atg16L1 in *Salmonella*-infected Caco-2 cells. Caco-2 cells were untreated (CON) or treated with fumonisin B1 (FB1) and then infected by *S. typhimurium* wild-type strain SL1344 for indicated times. Immunoblots were performed on membrane lysates with antibody to detect Atg16L1 and NOD2expression, and E-cadherin for normalization of membrane protein. Representative immunoblots are shown.
10.1186/s13099-016-0088-2 Effect of fumonisin B1on *Salmonella*-induced hBD-2 mRNA expression in Caco-2 cells. Caco-2 cells were left untreated, or treated with 25 μg/mL fumonisin B1 (FB1). They were then infected with the wild-type *S. Typhimurium* strain SL1344 for 1 h. Total RNA was prepared and analyzed by real-time quantitative PCR to estimate amounts of hBD-2 transcript. The amount of hBD-2 mRNA expression, normalized to the corresponding amount of GAPDH transcript, is shown as the fold increase over uninfected, control cells (CON). Results are represented as mean ± S.E.M. for at least three determinations from independent experiments. An asterisk indicates a significant difference (*p* < 0.005).

## Abbreviations

NOD2: nucleotide-binding oligomerization domain-containing protein 2
ATG16L1: autophagy-related protein 16-like 1
hBD-2: human beta-defensin-2
IEC: intestinal epithelial cell
PI3K: phosphatidyl-inositol-3-kinase
SCV: *Salmonella*-containing vacuole
