# Supplementary figures and images for: De Novo sphingolipid synthesis is essential for Salmonella-induced autophagy and human beta-defensin 2 expression in intestinal epithelial cells
Source: Gut Pathog. 2016 Feb 18;8:5. doi: 10.1186/s13099-016-0088-2 (PMC4758167; doi:10.1186/s13099-016-0088-2)

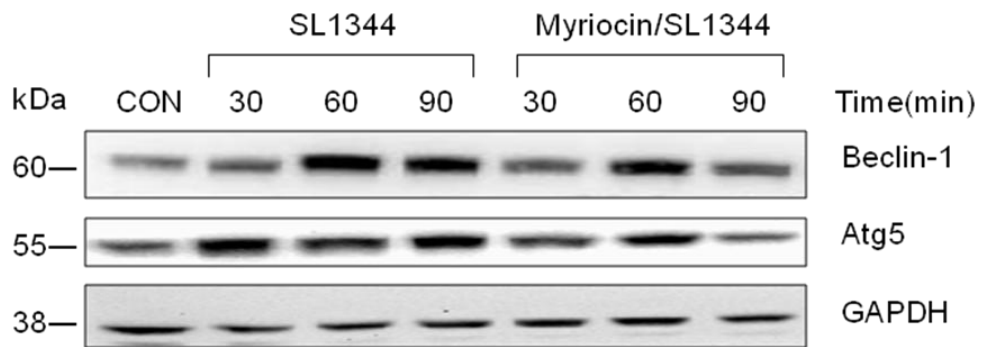

Fig. S1A

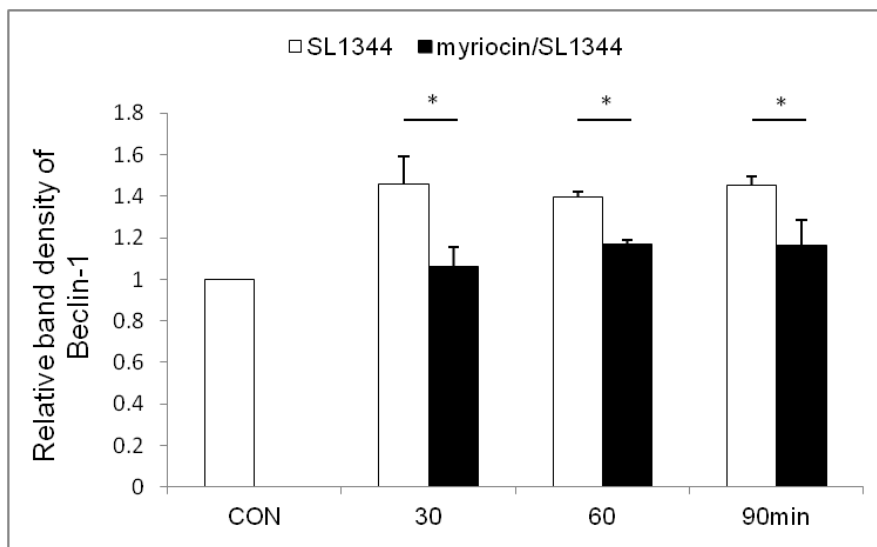

Fig. S1B

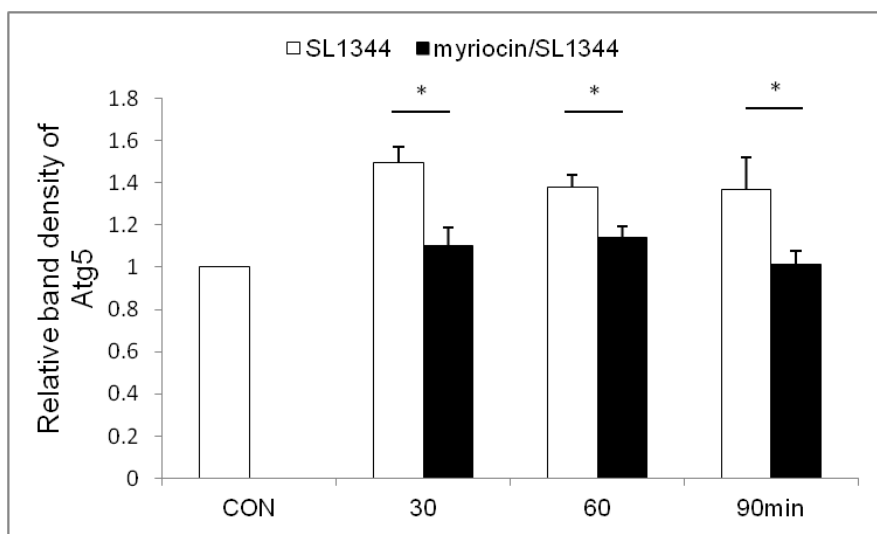

Fig. S1C

Supplement: Supplementary file 1 — 10.1186/s13099-016-0088-2 Effect of myriocin on the expression of autophagy in Salmonella-infected SW480 cells. SW480 cells were untreated (CON) or treated with myriocin and then infected by S. typhimurium wild-type strain SL1344. Immunoblots were performed on whole cell lysates with antibody to detect autophagy Beclin-1 and Atg5 proteins expression, or GAPDH for normalization of proteins. Representative immunoblots (A) and densitometric quantification of immunoreactive bands are shown. The relative band intensities of Beclin-1 (B) and Atg5 (C) in untreated (white) and treated (black) SW480 cells are quantified as fold increases compared with the control cells. Each value represents the mean ± S.E.M. of 3 independent experiments. An asterisk indicates a significant difference (p < 0.05). [file 13099_2016_88_MOESM1_ESM.pdf]

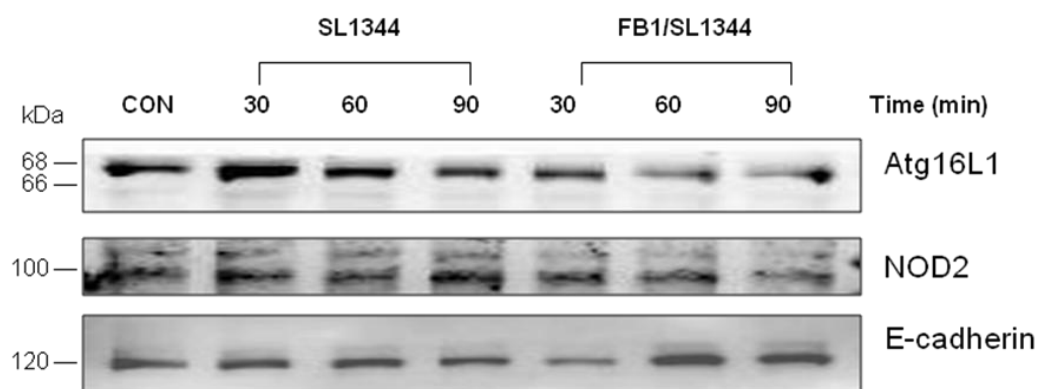

Fig. S2

Supplement: Supplementary file 2 — 10.1186/s13099-016-0088-2 Effect of fumonisin B1 on the membrane recruitment of NOD2 and Atg16L1 in Salmonella-infected Caco-2 cells. Caco-2 cells were untreated (CON) or treated with fumonisin B1 (FB1) and then infected by S. typhimurium wild-type strain SL1344 for indicated times. Immunoblots were performed on membrane lysates with antibody to detect Atg16L1 and NOD2expression, and E-cadherin for normalization of membrane protein. Representative immunoblots are shown. [file 13099_2016_88_MOESM2_ESM.pdf]

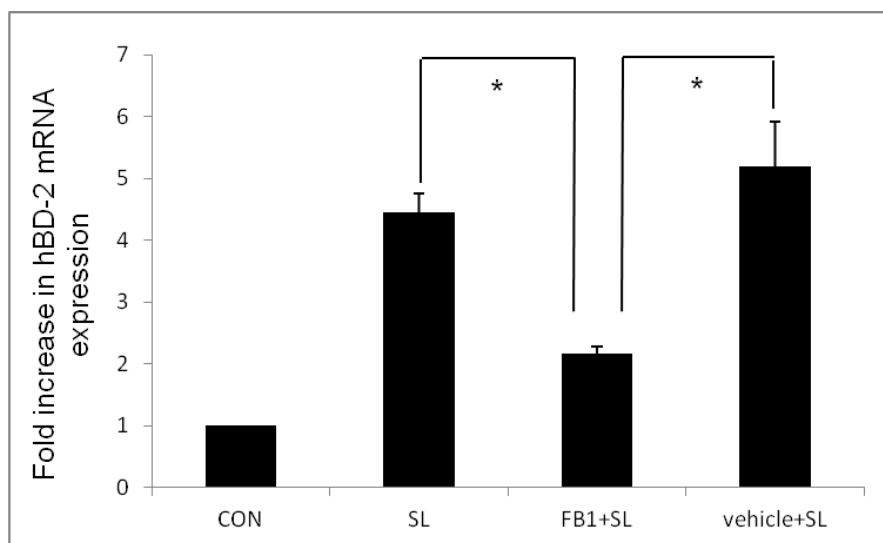

Fig. S3

Supplement: Supplementary file 3 — 10.1186/s13099-016-0088-2 Effect of fumonisin B1on Salmonella-induced hBD-2 mRNA expression in Caco-2 cells. Caco-2 cells were left untreated, or treated with 25 μg/mL fumonisin B1 (FB1). They were then infected with the wild-type S. Typhimurium strain SL1344 for 1 h. Total RNA was prepared and analyzed by real-time quantitative PCR to estimate amounts of hBD-2 transcript. The amount of hBD-2 mRNA expression, normalized to the corresponding amount of GAPDH transcript, is shown as the fold increase over uninfected, control cells (CON). Results are represented as mean ± S.E.M. for at least three determinations from independent experiments. An asterisk indicates a significant difference (p < 0.005). [file 13099_2016_88_MOESM3_ESM.pdf]
